# Addendum to: Murre (2021). The Godden and Baddeley (1975) experiment on context-dependent memory on land and underwater: a replication

**DOI:** 10.1098/rsos.211924

**Published:** 2022-01-05

**Authors:** Jaap Murre

**Affiliations:** Department of Psychology, University of Amsterdam, PO Box 95195, 1001, Amsterdam, The Netherlands

**Keywords:** context-dependent memory, replication study, divers, corrigendum

## Abstract

The analysis in Murre (2021) should have been done with a repeated measures ANOVA with an order effect as a between-subjects covariate. A reanalysis reinforces the conclusion that this study was not a successful replication of Godden & Baddeley (1975).

*R. Soc. open sci*. **8**, 200724 (Published Online 3 November 2021) (doi:10.1098/rsos.200724)

## 1. Description

In the Godden & Baddeley replication, I aimed to replicate the original analyses as closely as possible [1]. The authors did not state in their paper that they did a repeated measures analysis or used covariates, so I concluded that I should not do this either. A detailed analysis by Sean Mackinnon on the basis of my online data files at OSF (https://osf.io/5xas9/), however, has made it clear that my conclusion was wrong and that I should have done a repeated measures ANOVA after all, also including an order effect as a between-subjects covariate. In that way, one arrives at the (1,12) degrees of freedom mentioned by Godden & Baddeley [1] for a sample size of *n* = 16.

Sean Mackinnon also spotted a minor error in condition *DD-Group 2-Subject 8* where the correct raw value is 13 but because of an error in the Excel sheet has been changed to 14. My analysis works with the value 14, this should have been 13.

## 2. Implication

The errors above do not turn my study into a successful replication in the expected direction. However, the Learn*Recall interaction now becomes statistically significant at a *p* of 0.017 (see file at OSF within.scuba.v2.pdf). Also, the Learn*Recall*Order interaction now just reaches statistical significance at *p* = 0.045 using the correct value of 13 (instead of 14).

## 3. Conclusion

The data as shown in Murre (2021) in figure 1*a* show a quite different pattern than what a successful replication should have shown (figure 1*b*). For example, the Dry–Dry condition and the Wet–Wet condition should have been high and the Dry–Wet and Wet–Dry conditions should have been low in figure 1*a*. The new analysis described above indicates that the pattern of results in figure 1*a* now presents a significant interaction. But this only reinforces the conclusion that this study was not a successful replication of Godden & Baddeley [1].
